# 25-year neuroimaging research on spoken language processing: a bibliometric analysis

**DOI:** 10.3389/fnhum.2024.1461505

**Published:** 2024-11-27

**Authors:** Yuxuan Zheng, Boning Zhang

**Affiliations:** ^1^School of Interpreting and Translation, Beijing International Studies University, Beijing, China; ^2^AI and Cognition Laboratory, Beijing International Studies University, Beijing, China; ^3^School of English Studies, Beijing International Studies University, Beijing, China

**Keywords:** neuroimaging, spoken language processing, bibliometric analysis, Alzheimer's disease, oscillations

## Abstract

**Introduction:**

Spoken language processing is of huge interest to cognitive and neural scientists, as it is the dominant channel for everyday verbal communication. The aim of this study is to depict the dynamics of publications in the field of neuroimaging research on spoken language processing between 2000 and 2024.

**Methods:**

A bibliometric analysis was conducted to probe this particular subject matter based on data retrieved from Web of Science. A total of 8,085 articles were found, which were analyzed together with their authors, journals of publication, citations and countries of origin.

**Results:**

Results showed a steady increase of publication volume and a relatively high academic visibility of this research field indexed by total citations in the first 25 years of the 21st century. Maps of frequent keywords, institutional collaboration network show that cooperations mainly happen between institutions in the United States, the United Kingdom and Germany. Future trends based on burst detection predict that classification, Alzheimer’s disease and oscillations are potential hot topics.

**Discussion:**

Possible reasons for the result include the aging of the population in developed countries, and the rapid growth of artificial intelligence in the past decade. Finally, specific research avenues were proposed which might benefit future studies.

## 1 Introduction

Neuroimaging techniques have been proved an extremely useful tool for linguistic research (*cf.* Gernsbacher and Kaschak, 2003), and have fruited interesting and important findings that furthered our knowledge of the neural underpinnings of language. For example, early neuroimaging studies showed that learning another language leads to the density increment of grey matter in the left inferior parietal cortex, which is mediated by language proficiency and age of acquisition (Mechelli et al., 2004), that the left posterior temporal lobe and the inferior parietal lobe are consistently activated during word retrieval (Warburton et al., 1996), and that post-stroke language recovery relies heavily on the right inferior frontal cortex (Perani et al., 2003). Such findings not only illuminated the quest for the exact brain structures that occasion language, but also bore practical significance regarding the treatment of aphasia. Neuroimaging research on language has now become even more vibrant as imaging techniques advance swiftly.

That notwithstanding, the landscape of neuroimaging research on language is not facile to depict due to the miscellanea within this discipline. There seems to be quite a few contrasting paradigms, if not dichotomies, in this research field, such as the temporal-versus-spatial technique selection (e.g., EEG vs. fMRI), the auditory -versus- articulatory mode of experimentation, the word-versus-sentence stimuli presentation, and the healthy-versus-clinical participant sampling. It is therefore necessary to narrow down the scope before any topic can be reviewed in the area of neuroimaging language research. As far as mode is concerned, research on language processing runs the whole gamut where one end is focused on auditory processing while the other attaches huge importance to articulatory processing. The current paper aims to present a panoramic view of neuroimaging research on spoken/articulatory language processing in the first 25 years of the 21st century across the spectrum of imaging techniques, irrespective of the stimuli selection and sampling population, using the method of bibliometric analysis.

In the field of neuroimaging research on spoken language processing, a model was proposed that attributes human speech production to a dorsal stream in the brain, involving such brain regions as inferior parietal and posterior frontal lobe, as opposed to a ventral stream responsible for speech comprehension that mainly taps the middle and inferior temporal cortices (Hickok and Poeppel, 2004, 2007). To solve this conundrum, different neuroimaging techniques have been employed to investigate auditory and articulatory language processing, including fMRI (Behroozmand et al., 2015; Correia et al., 2020; Hickok et al., 2003; Lee et al., 2012; Riecker et al., 2005), PET (Dufor et al., 2007; Josephs et al., 2010; Schulz et al., 2005; Utianski et al., 2018; Vanlancker-Sidtis et al., 2003), EEG (Antons et al., 2012; Fiedler et al., 2017; Hausfeld et al., 2012; Kühnis et al., 2013; Zoefel and VanRullen, 2016), fNIRS (Defenderfer et al., 2021; Lawrence et al., 2018; Walsh et al., 2017; Zhang et al., 2018) etc. Although some studies found that the processing of auditory and spoken language (or overt articulation) activates overlapping brain structures (Papathanassiou et al., 2000), both on the word level and on the sentence level (Price, 2012; Tremblay et al., 2013), the preponderance of evidence seems to indicate separate systems for speech perception and production.

As speaking is an oral activity consisting of tightly coordinated stages (Levelt, 1989), imaging evidences abound that reveal different mechanisms underlying these stages. For instance, it has been shown that speech motor control relies on the caudate nucleus-cerebellum circuit modulated by phonological complexity (Sörös et al., 2006), while the planning for articulation involves the left precentral gyrus of the insula (Dronkers, 1996). In another aspect, neuroimaging findings show that the neural underpinnings of speech production are modulated by diverse linguistic traits. On the word level, the anterior cingulate cortex subserves the monitoring of ongoing word production (Christoffels et al., 2007), and non-word repetition of a novel language incurs the extra activation of the inferior frontal gyrus and the left anterior insula, as compared to the repetition of native language (Moser et al., 2009). The rate of syllabic-level speech production is mirrored in the activation of such motor brain structures as the cerebellum, the primary motor cortex and the thalamus (Riecker et al., 2005; Wildgruber et al., 2001). On the sentence level, compared to jaw and tongue movements, self-initiated sentential speech activates the superior temporal gyri in both hemispheres, and in particular, production of propositional speech relative to non-propositional speech elicits extensive medial and lateral activities (Dhanjal et al., 2008). Geranmayeh et al. (2014) went a step further to try to distinguish overt production of propositional speech from other tasks such as counting and decision with fMRI. Their experiments revealed a left lateralized fronto-temporal–parietal network unique to sentential speech production, partly echoing their previous findings comparing speech and tongue movement (Geranmayeh et al., 2012). Moreover, compared with monolinguals, speaking English as L2 has been found to correlate with increases in fractional anisotropy in more posterior left hemisphere white matter regions (Kuhl et al., 2016). Clinically, neuroimaging research has also fruited key findings for the understanding of the pathology of stuttering (*cf.* Garnett et al., 2019) and aphasia (Fridriksson et al., 2012; Thompson and den Ouden, 2008). For example, experiments on post-acute aphasia patients using CT and MRI showed that the traditional dorsal speech production route could be extended further, covering more specific brain structures from the supramarginal gyrus through inferior postcentral and precentral sensorimotor regions to premotor cortical regions (Mirman et al., 2015). Given the many findings above, it is not difficult to summarize that the neural mechanisms of spoken language processing are a huge network where diversity outstrips commonality, although some brain structures, such as the superior temporal gyri, have been repeatedly found to be activated across different spoken language experiments. Such disparity might result from different techniques and designs between studies, and calls for further exploration.

Bibliometric analysis (BA) is a quantitative method that comprehensively evaluates the quality of academic journals, the contribution of researchers, the collaboration between institutions and/or countries etc., using statistics such as citations, and software such as CiteSpace (Chen, 2014). BA can reveal not only existing patterns of academic publications in a research field, but also the prospective trends in that discipline. BA has been widely employed to assess linguistic studies (e.g., Guo, 2022; Sun and Lan, 2021; Villalobos et al., 2022; Zhang, 2020) though, it is surprising to know that lacunae of mode-specific BA stand out in the realm of linguistic research, i.e., there is no BA of neuroimaging research on spoken and visual language processing, respectively, in existing literature. This necessitates and justifies the current analysis. Specifically, the current research aims to answer the following three questions: (1) what was the general trend in the first 25 years after 2000 with respect to the publications of neuroimaging research on spoken language processing? (2) what was the collaboration dynamics in this field? (3) what is projected to be the buzzwords in the future?

## 2 Materials and methods

### 2.1 Study design and data selection

Neuroimaging relevant literature on spoken language processing data were retrieved from Web of Science Core Collection (WoSCC), which is one of the most commonly used database and could provide reliable information for bibliometric analysis (Falagas et al., 2008). Four editions were selected including the Science Citation Index Expanded (SCI-expanded), the Social Sciences Citation Index (SSCI), the Arts & Humanities Citation Index (AHCI), and the Emerging Sources Citations Index (ESCI), as neuroimaging language studies are highly interdisciplinary. The query was limited to the topic field, including search title, abstract, author keywords, and keywords plus. The search terms were as follows: (Brain imaging OR neuroimaging OR MRI OR magnetic resonance imaging OR EEG OR electroencephalogram OR MEG OR Magnetoencephalography OR fNIRS OR Functional near-infrared spectroscopy OR PET OR Positron emission tomography OR DTI OR Diffusion tensor imaging OR CT OR computed tomography OR ERP OR event related potential OR Single-photon emission computed tomography OR SPECT OR Diffuse optical imaging OR DOI) AND (Language OR Linguistics) AND (Oral OR Speak OR Spoke OR Speech).

In addition, to make results more precise, the search was limited to the neuroimaging relevant subjects. The publication language was limited to English, and publication type was limited to original articles and reviews. All the searches were conducted on 24 Oct, 2024 with an initial time from 2000. Publication data were exported as “Plain Text File” with the “full record and cited references” for analysis in bibliometric software.

### 2.2 Data analysis

**Figure 1:**
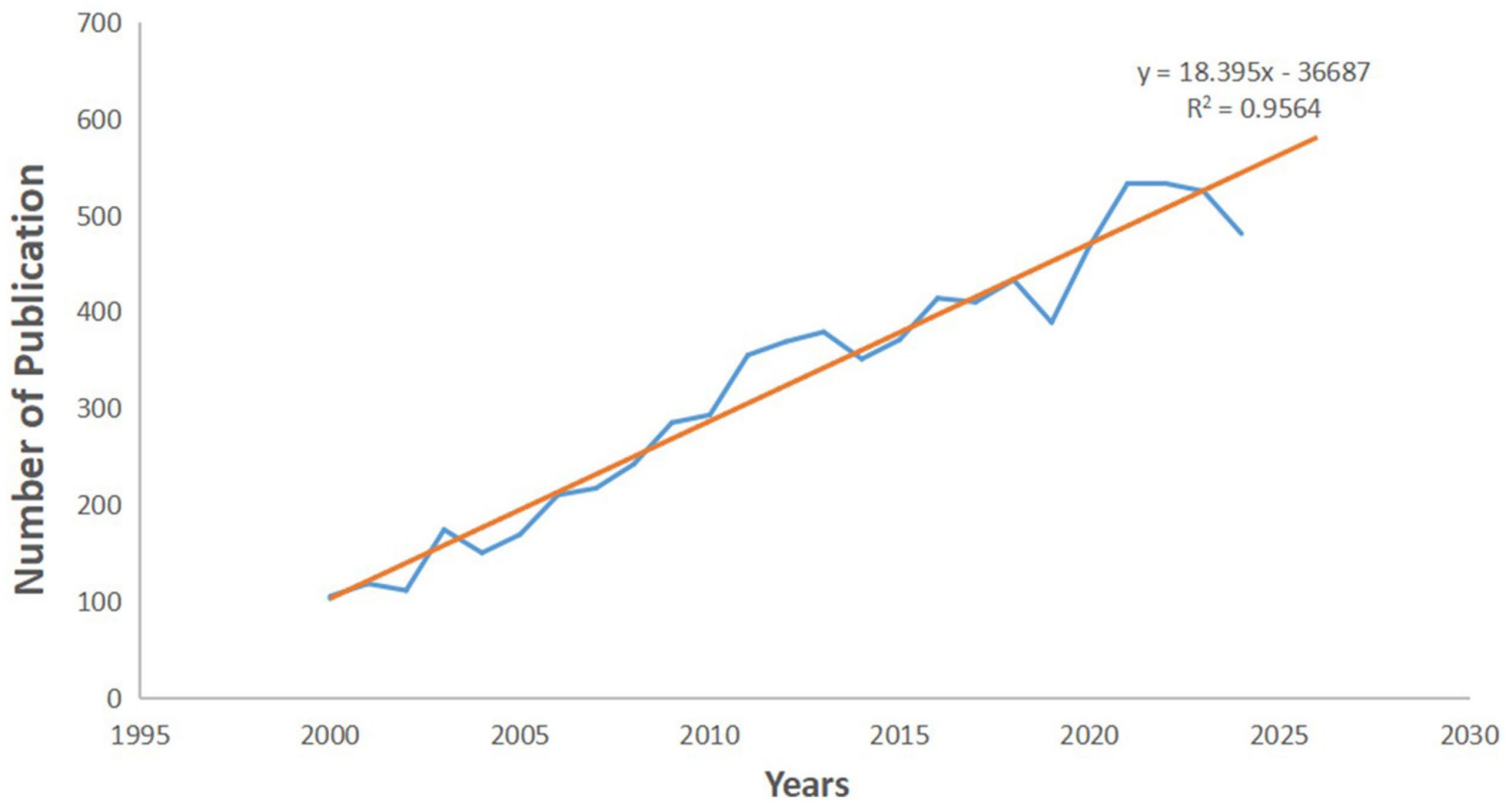
Number of publications by year (blue dot-dash line) and the trend of publication (orange line).

**Figure 2:**
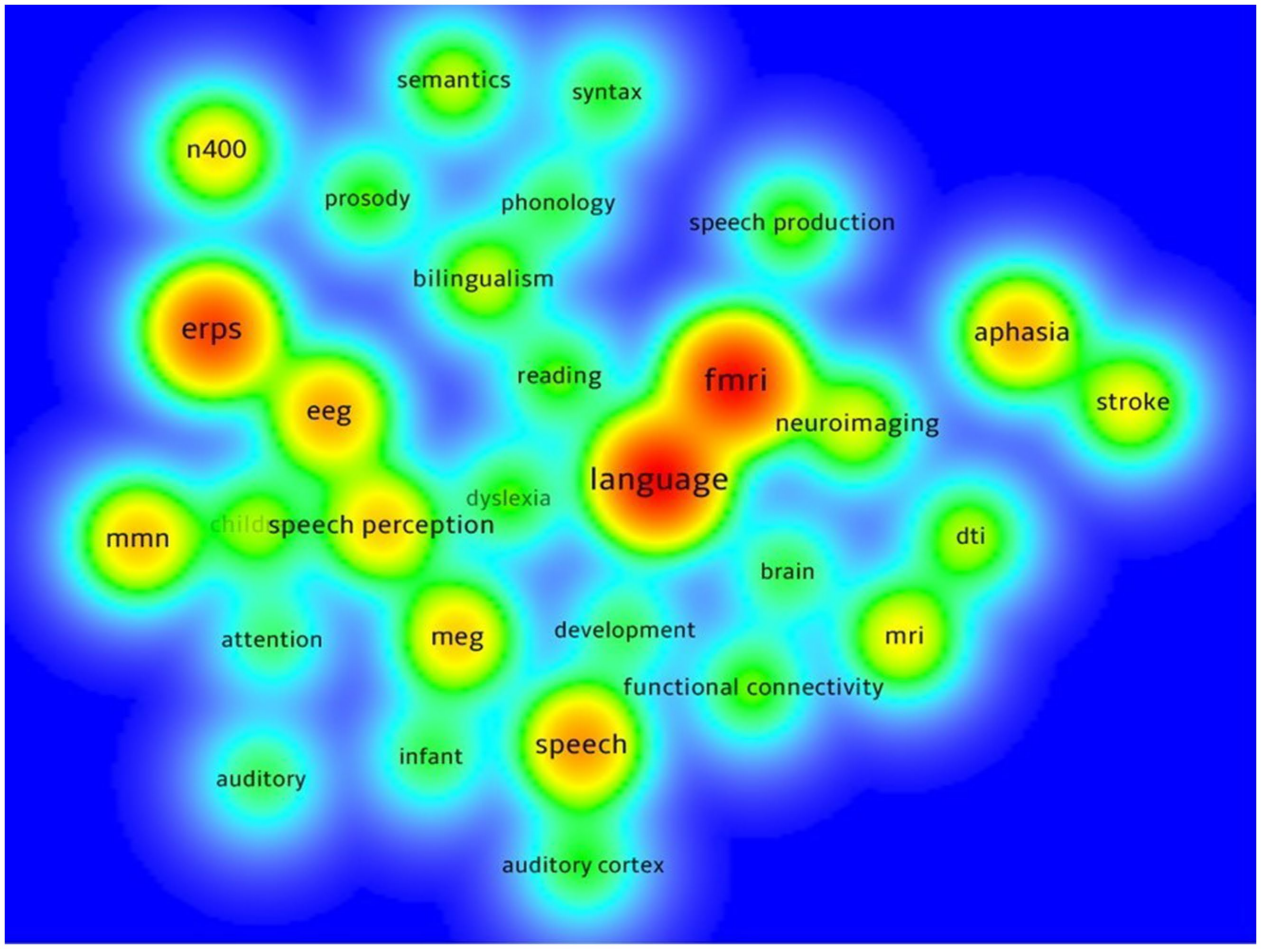
Density visualization of keywords co-occurrence.

**Figure 3:**
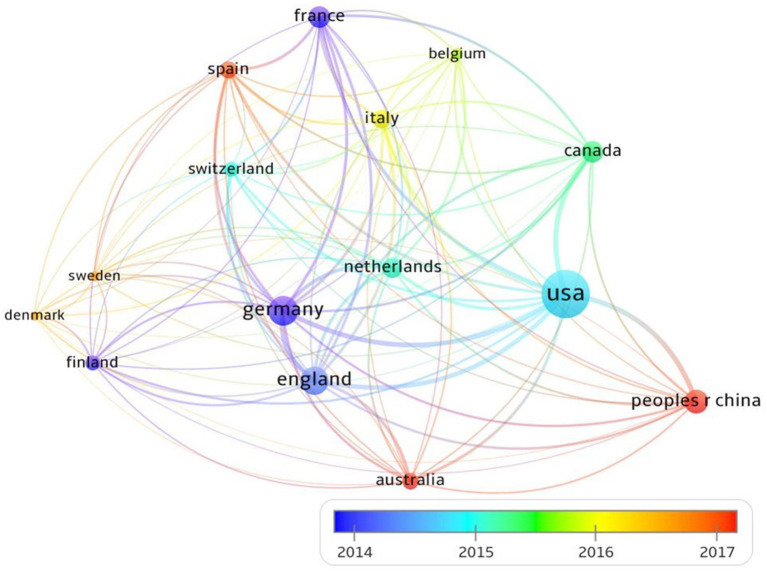
Overlays visualization of country collaboration of neuroimaging research on spoken language processing (2014-2017).

**Figure 4:**
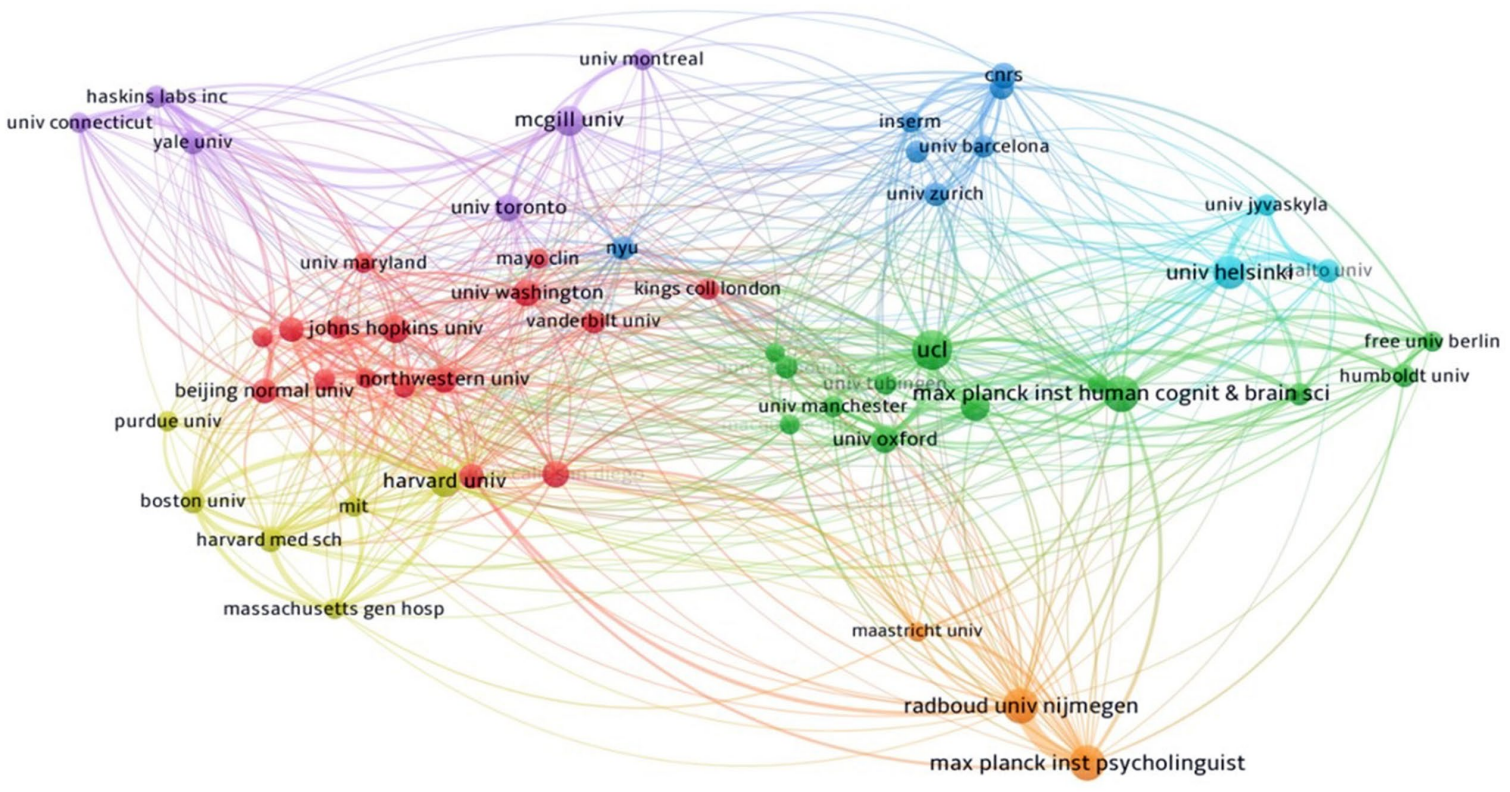
Network visualization of institutional collaboration in the field of neuroimaging research on spoken language processing.

**Figure 5:**
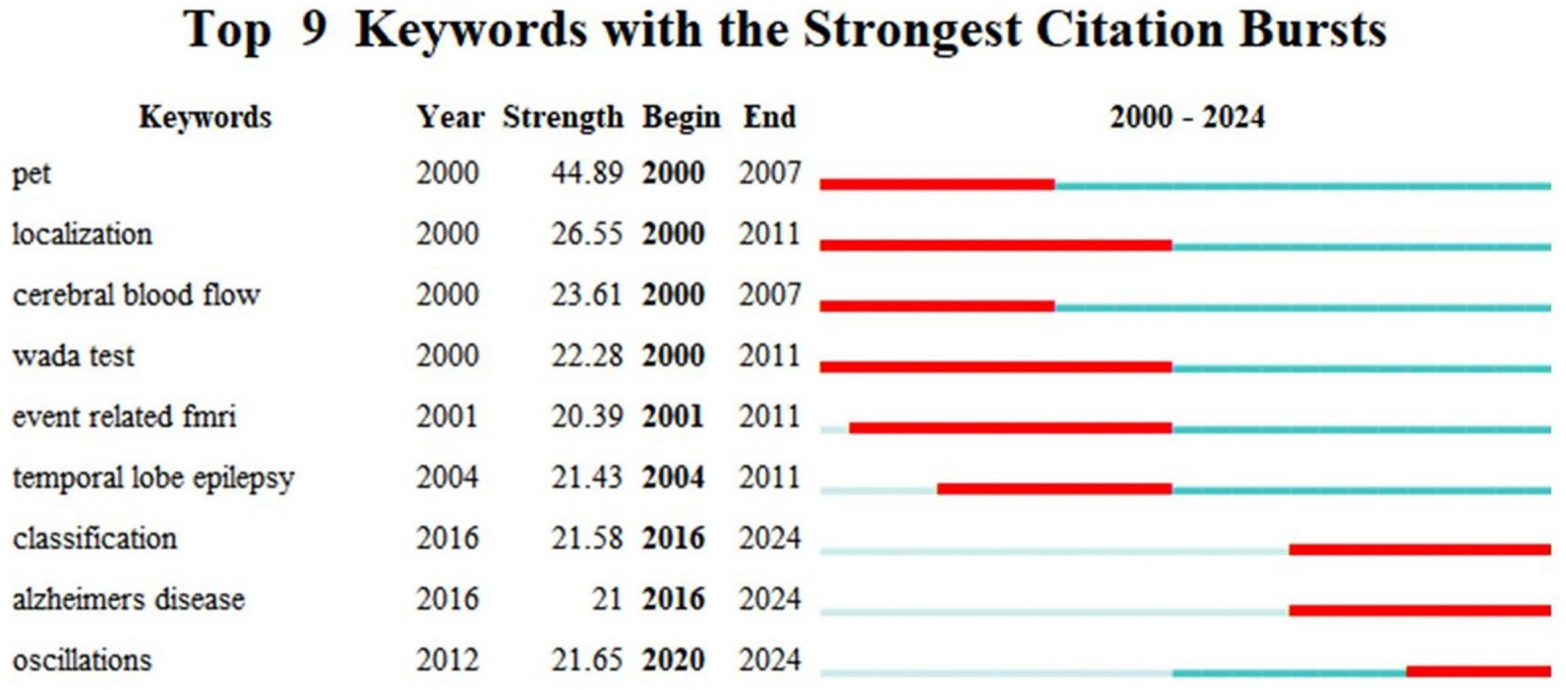
Top 9 keywords with the strongest citation bursts in neuroimaging research on spoken language processing generated by Citespace.

**Figure 6:**
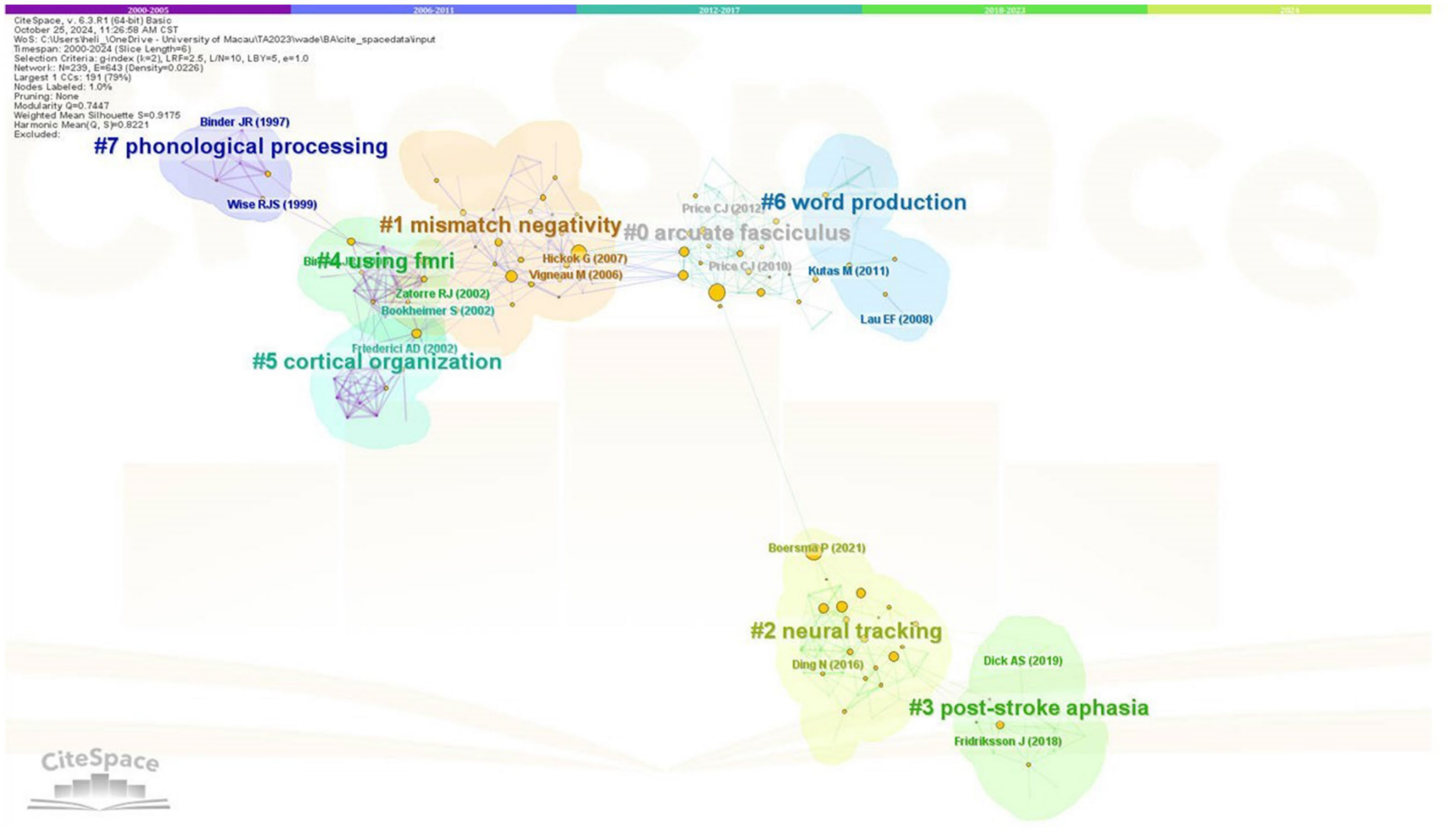
Results of the cluster-based document co-citation analysis (DCA).

Text files extracted from Web of Science were then imported into three bibliometric programs in order to examine the development and status of relevant research. Biblioshiny is a widely used web interface based on R program to conduct bibliometric analysis, which extracts the features of publications across four different level metrics: countries, journals, institutions, and authors. First, raw data (in zip format) were imported into Biblioshiny. Then the raw data were filtered with default values, in agreement with those in the initial data selection stage. After that, Biblioshiny returned results of annual scientific production (shown in Figure 1) and most global cited documents (shown in Table 1) from 2000 to 2024. VOSviewer is another program to construct and view bibliometric maps (van Eck and Waltman, 2010), and could generate maps of institutions and countries based on co-authorship data, as well as maps of keywords based on co-occurrence data. In VOSviewer, a map based on bibliographic data was created by selecting “co-occurrence” and “author keywords” as the analysis types and “full counting” as the counting method. Then the minimum number of co-occurrence of a keyword was set to be 25 for thresholding. Altogether 171 keywords were above this threshold and thus were kept. For each of the 171 keywords, the total strength of the cooccurrence links with other keywords was calculated. The keywords with the greatest total link strength were kept. Finally, 30 keywords were selected for visualization (shown in Figure 2). Such procedure was then repeated for producing collaboration results, with “co-authorship” and “countries” replacing “co-occurrence” and “author keywords” respectively in the parameter setting. Forty documents were chosen as the inclusion threshold for a country to appear on the collaboration map, and a total of 15 countries were finally visualized (shown in Figure 3). Lastly, the same procedure was applied to producing the institutional collaboration map, with the only difference being the parameter “organizations” rather than “countries.” As a result, 50 institutions were included in the map (shown in Figure 4). CiteSpace is a bibliometric program for calculating references with the strongest citation bursts. In CiteSpace, data were categorized according to the co-occurring author keywords and keywords plus, and the k value in the g-index was set as 2 to extract a moderate size network. Finally, results of keywords with the strongest citation bursts and document co-citation analysis were visualized (shown in Figures 5, 6 respectively).

**Table 1:** Top 10 most-cited papers in the area of neuroimaging research on spoken language processing from 2000 to 2024.

SCR	Title (*N* = 6,608)	Journal	FA	TC	TC/Y	Year
1	Imaging cognition II: An empirical review of 275 PET and fMRI studies	Journal of Cognitive Neuroscience	Roberto Cabeza	2,725	109.0	2000
2	Thirty years and counting: finding meaning in the N400 component of the event-related brain potential (ERP)	Annual Review of Psychology	Marta Kutas	2,750	196.43	2011
3	Dorsal and ventral streams: a framework for understanding aspects of the functional anatomy of language	Cognition	Gregory Hickok	1,513	72.05	2004
4	Towards a neural basis of auditory sentence processing	Trends in Cognitive Sciences	Angela D Friederici	1,391	60.48	2002
5	Perisylvian language networks of the human brain	Annals of Neurology	Marco Catani	1,339	66.95	2005
6	The spatial and temporal signatures of word production components	Cognition	Peter Indefrey	1,359	64.71	2004
7	A review and synthesis of the first 20 years of PET and fMRI studies of heard speech, spoken language and reading	NeuroImage	Cathy J Price	1,419	109.15	2012
8	Meta-analyzing left hemisphere language areas: Phonology, semantics, and sentence processing	NeuroImage	Mathieu Vigneau	1,312	69.05	2006
9	Meta-analysis of the functional neuroanatomy of single-word reading: method and validation	NeuroImage	Peter E Turkeltaub	1,212	52.70	2002
10	Maps and streams in the auditory cortex: nonhuman primates illuminate human speech processing	Nature Neuroscience	Josef P Rauschecker	1,196	74.75	2009

SCR, standard competition ranking; FA, first author; TC, total number of citations; TC/Y, total number of citations per year.

## 3 Results

A total number of 8,085 articles were retrieved spanning the first 25 years of the 21st century. Figure 1 shows the dynamics of total annual publications over the period of 2000–2024, and the projected volume till 2030 based on a linear model (the orange line in Figure 1). The annual publication curve presents a roughly spiral growth of research production from 2000 to 2024, and the number of total publications across such period almost quadrupled. The linear fitting has a very high explanatory power for the data with an *R*^2^ value of 0.956. According to the linear model, there will be over 500 publications in the area of neuroimaging research on spoken language processing in 2025, almost 5 times more than the number in 2000, indicating a lasting heat on this specific research branch.

Table 1 lists the top 10 most cited academic papers on the topic of neuroimaging research of spoken language processing, with relevant information of the publication such as the source journal, first author and number of total citations. First, all of the 10 articles have been cited for at least 1,100 times, showing the vast interest aroused by the topic of neuroimaging spoken language processing. *NeuroImage* is the journal that publishes the most number of highly cited papers, followed by *Cognition*. Clearly, most of the highly-cited papers are review articles. Eight of the 10 papers are published before 2010, and no article after 2012 enters the top 10 most-cited list, which might suggest that this research avenue lacks substantial breakthrough after 2012.

Figure 2 is a density visualization that presents the most frequently used keywords in neuroimaging research on spoken language processing, the warmer the color, the higher the frequency. First, as an umbrella term, language is the most frequently used keyword in the existing research literature, accompanied by fMRI in one common co-occurrence network that also includes neuroimaging. Following that is the second most popular keyword cluster of ERPs, together with EEG and speech perception that forms another relatively large co-occurrence network. Obviously, the second and third clusters are technique-related keyword networks. Ranking third is the word speech with some peripheral words such as development and auditory cortex. Other smaller clusters are dominated by such keywords as aphasia, mmn (mismatch negativity) and N400. Results of the keywords cooccurrence analysis demonstrate that among all imaging techniques fMRI is the most popular one, and that neuroimaging spoken language research has been implemented more on clinical populations than on healthy participants.

Figure 3 portrays the contribution by and collaboration between different countries (top 15) over the period of 2014–2017 in the field of neuroimaging research on spoken language processing. The size of the node represents the production volume of a country, while the color of the node indicates the time of publication from that country. In terms of total publication frequencies, USA comes first with 3,181 publications, followed by Germany (1179) and UK (1041). Other countries with over 500 publications include China, France, Netherlands and Canada. With respect to the time of publication, countries such as UK, USA and Germany have had a relatively longer history of neuroimaging research on spoken language processing, whereas China, Australia and Spain are newly into this field although all have produced sizeable amount of articles. USA is the hub for academic collaborations with 1,550 internationally co-authored publications. Note that the time line only shows the collaboration dynamics between 2014 and 2017 in order to achieve the best contrastive visual effect. When the timeline was stretched to cover a 5-year period anywhere between 2000 and 2024, the contrast as manifested by line colors would be washed out, indicating an interesting fact that cooperations in this field saw intensive new dynamics between 2014 and 2017 and were in a quite stable state either before 2014 or after 2017.

Figure 4 illustrates the academic cooperation between global institutions. The whole collaboration network is characterized by seven clusters. The green cluster dominated by University College London and Max Planck Institute for Human Cognitive and Brain Sciences shows the cooperation mainly between British and German academic institutions, with a fairly large share of contributions from other universities in these two countries such as Oxford University and Humboldt University. As is shown by the red and yellow nodes, US universities form two big clusters characterized by more domestic coordination than international partnership, albeit some connections with Chinese institutions (e.g., Beijing Normal University) and British ones (e.g., King’s College London). In contrast, Canadian universities marked with purple nodes maintained extensive relations with other institutions across continents. There is also a small orange cluster consisting of three Dutch institutions, i.e., Radboud University Nijmegen, Max Planck Institute for Psycholinguistics, and Maastricht University. It seems that Dutch academic units are a bit more self-reliant than their counterparts in other countries, especially Canada. Lastly, there are two minor networks showing the collaboration of Finnish, Spanish and Swiss universities.

Figure 5 shows the top 9 keywords that have had a surging appearance during a specific period of time between 2000 and 2024 using burst detection. It is conspicuous that in the early noughties research was characterized by specific neuroimaging techniques like PET, fMRI and Wada test, together with such explanatory terms as localization and cerebral blood flow. Among them, localization had the longest and most enduring popularity spanning the period of 2000–2011. After that the research hotspot transitioned to temporal lobe epilepsy, a common brain disease in the neuroimaging research literature. From 2016 onwards, the concept of classification, Alzheimer’s disease and oscillations attracted academic attention from the neuroimaging spoken language research community. In particular, oscillations showed a very high citation strength since 2020, still trending and are projected to be intriguing topics in the future.

Figure 6 shows the result of document co-citation analysis (DCA) across the first quarter of the 21st century. Altogether 8 co-citation clusters were identified, the largest of which was labelled arcuate fasciculus (cluster 0), followed by mismatch negativity (cluster 1). Less strong but more recent were the themes of neural tracking (cluster 2) and post-stroke aphasia (cluster 3), which were also situated in the farther position on the DCA map, indicating a relatively high independence of such themes. After that clusters 4, 5 and 6 were closely connected to clusters 1 and 2, with theme labels of using fMRI, cortical organization and word production, respectively. Lastly, cluster 7 reflected the early co-citation network under the theme of phonological processing, which was not so strong perhaps due to obsolescence. The modularity *Q* = 0.745 and silhouette S = 0.918 show that the clustering has very high validity and reliability.

## 4 Discussion

A bibliometric analysis of the first 25 years of neuroimaging research on spoken language processing was implemented based on data retrieved from Web of Science. Results show that the total number of publications in this area has been growing steadily and will continue to grow until 2030, that it has a relatively high academic visibility with its top 10 papers all having over 1,100 citations, that research in this area is largely implemented with fMRI and EEG, that academic collaborations mainly happen among European, North American and East Asian countries/institutions, and that potential hot topics in the future may include such keywords as classification, Alzheimer’s disease and oscillations.

The reason behind the publication boom from 2000 to 2024 might be threefold. In a sense speaking takes precedence over writing in linguistics (for a comprehensive review on the relationship between written and spoken language *cf.* Olson, 1996), and therefore spoken language processing is of great interest to linguists, as evidenced by the fact that early attempts which gave birth to the discipline of neurolinguistics were all based on spoken language processing (e.g., Broca, 1861; Wernicke, 1874). The fast development and the wider application of various neuroimaging techniques in academic research in the 21^st^ century accounts for another big share of publication increase in neuroimaging research on spoken language processing (*cf.* Scott, 2021). Lastly, the mode of spoken language processing has clinical significance, i.e., speaking has been included in test batteries or used as a screening method for diseases of various kinds, such as stroke (e.g., Breier et al., 2008; Ilves et al., 2014; Sreedharan et al., 2019; Sreedharan et al., 2020), Parkinson’s disease (Dick et al., 2018; Hyder et al., 2021; Lee and Van Lancker Sidtis, 2020), Alzheimer’s disease (Deters et al., 2017; Pistono et al., 2021; Radanovic et al., 2009) and autism spectrum disorder (Arnold et al., 2009; Ghaziuddin and Gerstein, 1996; Volden, 2004), among both young populations (Jarrold et al., 2013; Sah and Torng, 2015; Wu et al., 2020) and senior participants (Rusz et al., 2013; Tjaden et al., 2013; Yorkston et al., 2017).

In terms of the most cited papers in this research area as listed in Table 1, the results are quite revealing as the papers with the most citations are also the most significant ones in pushing forward the understanding of the neural mechanisms of spoken language processing. For example, Kutas and Federmeier (2011) disentangled the many functions of the N400 ERP component in language processing, and reorganized previous empirical findings with a clear categorization that includes discourse processing, predictive processing, word recognition, bilingualism, semantic memory, recognition memory etc., providing a comprehensive framework for later studies to refer to. Similarly, an early review by Friederici (2002) has been cited for over a thousand times for its ground-breaking proposal of the neural basis for auditory sentence processing that mainly involves the bilateral temporo-frontal network including the left anterior STG for syntactic processing, the left MTG for semantic processing and the right posterior STG for prosodic processing. In a word, the top 10 most cited papers are also the most influential ones in this research avenue, in a sense confirming the effectiveness of citation analysis. Note however, the highly cited papers in the table are all review articles and no empirical or methodological paper was present. This could be a BA-induced bias, and warrants caution in terms of generalization of the analysis.

Another notable point in the results is the prediction that classification, Alzheimer’s disease and in particular, oscillations, are the trending topics in the research domain of neuroimaging experiments on language. The three trending topics are intertwined to some extent, with oscillations lying in the central position. First of all, classification is a process of obtaining specific EEG features after artifact removal and feature extraction, for the purpose of automatic application of EEG signals in practical settings such as brain-computer interface (BCI) (Craik et al., 2019). The rising popularity of the buzzword classification to some degree was occasioned by the booming development of artificial intelligence in the past decade, as manifested by the many deep learning algorithms and approaches employed in EEG classification (*cf.* He et al., 2021; Rabcan et al., 2020). Additionally, the trending of classification also echoes cluster 2 (neural tracking) on the DCA map, as the former can be a means to the latter (Lotte et al., 2018). Second, the mounting population of senior citizens in developed countries gave rise to the rapid growth of Alzheimer’s disease in recent years, the prediction and modeling of which is closely connected with neural oscillations (Goriely et al., 2020; Jafari et al., 2020). In fact, neural oscillations has been found to be a crucial index of a broader range of brain diseases (Weiss et al., 2023), which is in line with the result of DCA (cluster 3 post-stroke aphasia that is trending now). Lastly, the emerging hot topic of oscillations is possibly propelled by two reasons. In one aspect, oscillations have long been identified as the moderator for cognition (Ward, 2003), in another they serve and parallel the current speech processing technology advancement (Poeppel and Assaneo, 2020).

The fruitful results aforementioned notwithstanding, one stream of research that has been quite popular yet not captured by the current analysis, is language studies simultaneously using two or more imaging techniques (such as synchronous EEG-fMRI and/or EEG-NIRs). Such multimodal approach is not only necessary but also feasible (Wallois et al., 2012). However, so far it has not been used in spoken language processing studies, possibly due to technical difficulties related to the imaging techniques. Therefore it is quite promising to combine temporal and spatial imaging techniques in spoken language processing studies in the future.

## 5 Limitations

Despite the many revealing networks of collaboration between authors, academic institutions and countries presented, the current research has several limitations. First, the field of neuroimaging research is heavily reliant on the financial conditions of an institution or a country, as a set of EEG equipment costs at least tens of thousands of dollars and that of fMRI, millions. It is therefore reasonable to find the preponderance of neuroimaging research literature was produced in developed countries, while developing and underdeveloped countries could not afford such facilities. Consequently, the inequality in accessing the imaging techniques led to the bias of country/institution production in the BA results. Second, the method of BA *per se* is not without flaws. The variability in the searching process, e.g., difference in the types of publications selected (i.e., journal articles, conference papers or other forms) and languages covered could result in missing data, hence influencing the final analysis. Lastly, different analyses on the same subject matter may yield different results due to the discrepancy of underlying algorithms (e.g., Kleminski et al., 2022), an example of which is the citation-based article ranking that does not present empirically transformative research. This also adds to the inconsistency and bias of BA results.

## 6 Conclusion

Bibliometric analysis of the first 25 years of academic journal publications in the field of neuroimaging research on spoken language processing presents that, with fMRI and EEG being the most popular research techniques, this field is right in the spotlight. It receives attention particularly from *NeuroImage*, and institutions in the United States, the United Kingdom and Germany. In the future more efforts are likely to be directed to probing such keywords as Alzheimer’s disease and oscillations.

## Data Availability

The datasets presented in this study can be found in online repositories. The names of the repository/repositories and accession number(s) can be found at: www.webofscience.com.
